# A Comparative Analysis of Clinical Symptoms and Modified Pouchitis Disease Activity Index Among Endoscopic Phenotypes of the J Pouch in Patients With Inflammatory Bowel Disease

**DOI:** 10.1093/crocol/otae045

**Published:** 2024-08-02

**Authors:** Shintaro Akiyama, Nathaniel A Cohen, Jacob E Ollech, Cindy Traboulsi, Tina Rodriguez, Victoria Rai, Laura R Glick, Yangtian Yi, Joseph Runde, Russell D Cohen, Kinga B Skowron, Roger D Hurst, Konstantin Umanskiy, Benjamin D Shogan, Neil H Hyman, Michele A Rubin, Sushila R Dalal, Atsushi Sakuraba, Joel Pekow, Eugene B Chang, David T Rubin

**Affiliations:** University of Chicago Medicine Inflammatory Bowel Disease Center, Chicago, IL, USA; University of Chicago Medicine Inflammatory Bowel Disease Center, Chicago, IL, USA; University of Chicago Medicine Inflammatory Bowel Disease Center, Chicago, IL, USA; University of Chicago Medicine Inflammatory Bowel Disease Center, Chicago, IL, USA; University of Chicago Medicine Inflammatory Bowel Disease Center, Chicago, IL, USA; University of Chicago Medicine Inflammatory Bowel Disease Center, Chicago, IL, USA; University of Chicago Medicine Inflammatory Bowel Disease Center, Chicago, IL, USA; University of Chicago Medicine Inflammatory Bowel Disease Center, Chicago, IL, USA; University of Chicago Medicine Inflammatory Bowel Disease Center, Chicago, IL, USA; University of Chicago Medicine Inflammatory Bowel Disease Center, Chicago, IL, USA; University of Chicago Medicine Inflammatory Bowel Disease Center, Chicago, IL, USA; University of Chicago Medicine Inflammatory Bowel Disease Center, Chicago, IL, USA; University of Chicago Medicine Inflammatory Bowel Disease Center, Chicago, IL, USA; University of Chicago Medicine Inflammatory Bowel Disease Center, Chicago, IL, USA; University of Chicago Medicine Inflammatory Bowel Disease Center, Chicago, IL, USA; University of Chicago Medicine Inflammatory Bowel Disease Center, Chicago, IL, USA; University of Chicago Medicine Inflammatory Bowel Disease Center, Chicago, IL, USA; University of Chicago Medicine Inflammatory Bowel Disease Center, Chicago, IL, USA; University of Chicago Medicine Inflammatory Bowel Disease Center, Chicago, IL, USA; University of Chicago Medicine Inflammatory Bowel Disease Center, Chicago, IL, USA; University of Chicago Medicine Inflammatory Bowel Disease Center, Chicago, IL, USA

**Keywords:** pouchitis disease activity index, endoscopic pouch phenotype, clinical symptoms

## Abstract

**Background:**

The modified pouchitis disease activity index (mPDAI) based on clinical symptoms and endoscopic findings is used to diagnose pouchitis, but validated instruments to monitor pouchitis are still lacking. We recently established an endoscopic classification that described 7 endoscopic phenotypes with different outcomes. We assessed symptoms and compared mPDAIs among phenotypes in inflammatory bowel disease (IBD).

**Methods:**

We retrospectively reviewed pouchoscopies and classified them into 7 main phenotypes: normal (*n* = 25), afferent limb (AL) involvement (*n* = 4), inlet involvement (*n* = 14), diffuse (*n* = 7), focal inflammation of the pouch body (*n* = 25), cuffitis (*n* = 18), and pouch-related fistulas (*n* = 10) with a single phenotype were included. Complete-case analysis was conducted.

**Results:**

One hundred and three IBD patients were included. The median mPDAI was 0 (IQR 0-1.0) in patients with a normal pouch. Among inflammatory phenotypes, the highest median mPDAI was 4.0 (IQR 2.25-4.75) in cuffitis, followed by 3.0 (IQR 2.5-4.0) in diffuse inflammation, 2.5 (IQR 1.25-4.0) in inlet involvement, 2.5 (IQR 2.0-3.5) in AL involvement, 2.0 (IQR 1.0-3.0) in focal inflammation, and 1.0 (IQR 0.25-2.0) in the fistula phenotype. Perianal symptoms were frequently observed in pouch-related fistulas (8/10, 80%) and cuffitis (13/15, 87%). Among patients with cuffitis, all had incomplete emptying (6/6, 100%).

**Conclusions:**

We correlated the mPDAI with the endoscopic phenotypes and described the limited utility of symptoms in distinguishing between inflammatory phenotypes. Further studies are warranted to understand which symptoms should be monitored for each phenotype and whether mPDAI can be minimized after pouch normalization.

## Graphical Abstract

**Graphical Abstract:**
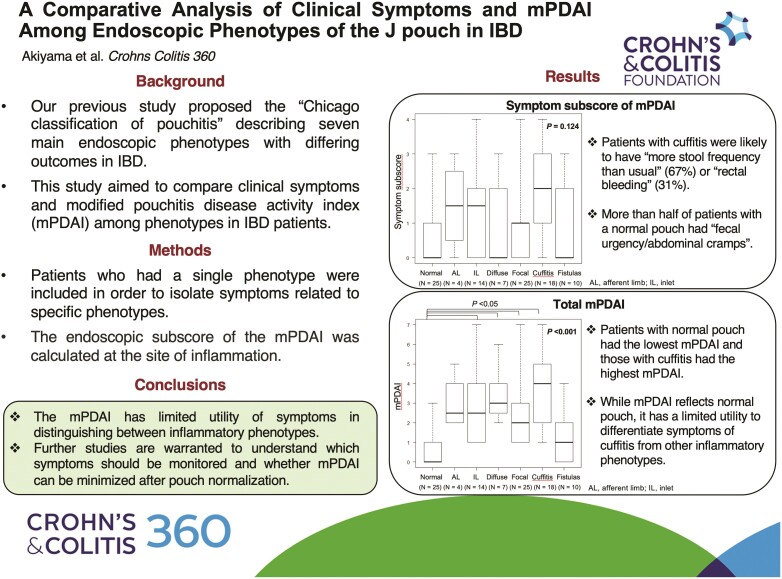

## Introduction

Restorative proctocolectomy with ileal pouch-anal anastomosis (IPAA) is a standard surgical approach for patients with inflammatory bowel disease (IBD). However, up to 70% of patients with IPAA can develop pouchitis, a novel inflammatory condition of this new anatomy.^1^ The pouchitis disease activity index (PDAI) was a novel scoring system developed to assess clinical symptoms, endoscopic inflammation, and acute histologic inflammation.^2^ The modified PDAI (mPDAI), in which the histologic score is omitted from the original PDAI, has similar sensitivity and specificity to the original PDAI in order to diagnose patients with acute pouchitis.^3^ The components of clinical symptoms in the mPDAI include stool frequency, rectal bleeding, fecal urgency or abdominal cramps, and fever >37.8 °C. However, these symptoms are not specific for patients with J pouches due to the variety of conditions that may contribute to such symptoms, including cuffitis,^4,5^ decreased pouch compliance/emptying,^5^ anastomotic stricture,^5^ irritable pouch syndrome,^6^ pelvic floor dysfunction,^5^ and jejunal bacterial overgrowth.^7^ Furthermore, the sensitivity of symptoms is poor, with many patients who have endoscopic inflammation of the pouch having no reported symptoms,^8^ suggesting that the mPDAI has a limited utility to diagnose pouchitis, and does not necessarily correlate with the severity and clinical course of pouchitis. Therefore, validated instruments to monitor disease activity of pouchitis remain to be developed and are needed.^9^

Although mPDAI includes a score of endoscopic inflammation, it does not take the distribution of pouch inflammation into account and lacks comments regarding the location of the lesion. Furthermore, the prognostic value of mPDAI to predict pouch outcomes has not been well described. To understand the complexity of inflammatory pouch conditions^10^ and their outcomes, our group previously assessed endoscopic findings according to the anatomical locations of the pouch and classified them into 7 main endoscopic phenotypes including (1) normal pouch, (2) afferent limb (AL) involvement, (3) inlet (IL) involvement, (4) diffuse inflammation of the pouch, (5) focal inflammation of the pouch, (6) cuffitis, and (7) pouch-related fistulas. We found that patients with a normal pouch or those with focal inflammation had favorable pouch outcomes, whereas patients with diffuse inflammation, inlet stenosis, and cuffitis had a significant increased risk of pouch excision over time.^11^ Our findings suggested that specific endoscopic inflammatory findings have value in prognosticating pouch outcomes.

We hypothesize that the ability of the mPDAI to reflect the severity of pouch inflammation can be improved by classifying symptoms in terms of the specific inflammatory phenotypes described in our classification. In this study, we assessed clinical symptoms in patients with a single pouch phenotype and then compared subscores and total scores of mPDAI among phenotypes to understand how mPDAI can vary between pouch phenotypes. Clinical symptoms not included in mPDAI were also assessed to identify additional candidates for disease activity monitoring in these patients.

## Materials and Methods

**Figure 1.**
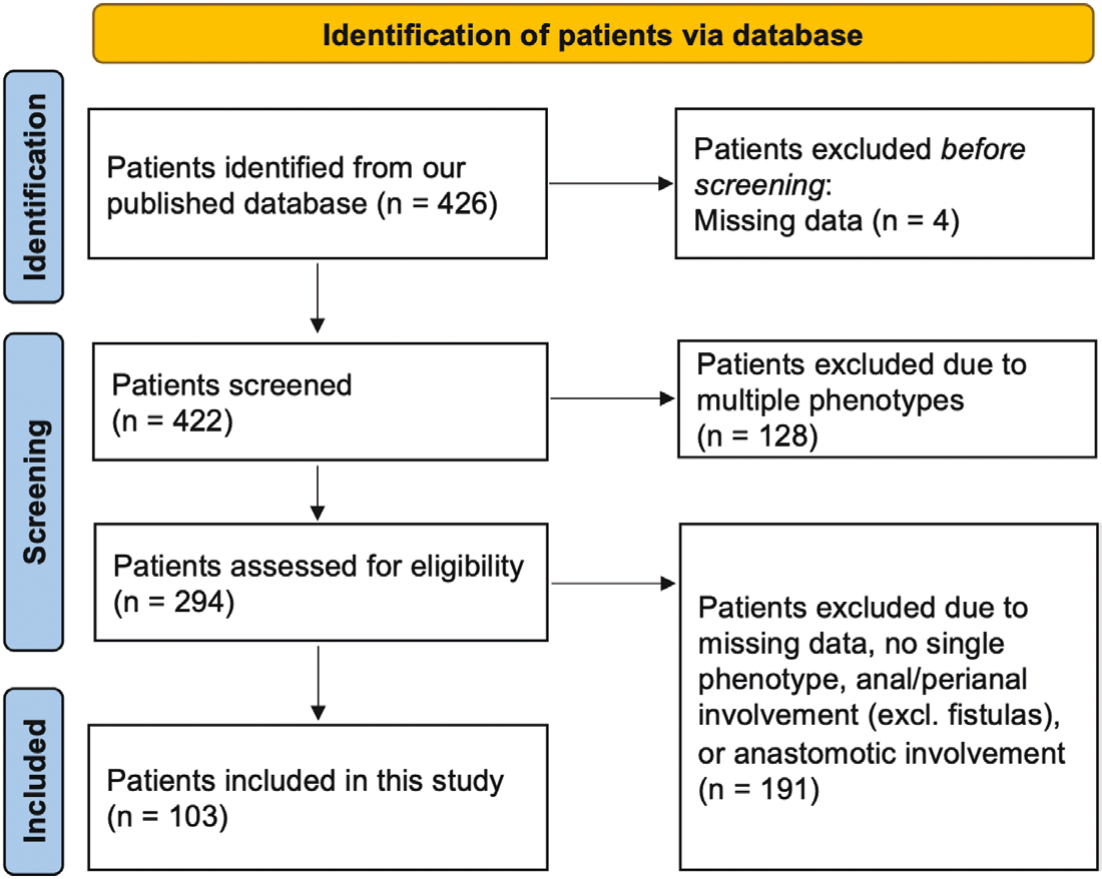
Flow diagram of the assessment of clinical symptoms and mPDAI in inflammatory bowel disease patients with a single endoscopic phenotype based on the Chicago classification in this study.

We performed a retrospective cohort study of IBD patients treated by total proctocolectomy with IPAA in J pouch configuration who subsequently underwent pouchoscopies at the University of Chicago between June 1997 and December 2019, using a previously described database of such patients.^11,12^ We have recently published data on endoscopic phenotype transition and created the dataset including the scope date when each phenotype was first identified.^12^ This dataset was used to identify patients with a single endoscopic phenotype (Figure 1).

### Data Collection

We retrospectively reviewed the endoscopic and medical records and the data collected are summarized in Supplementary Table S1.

In terms of endoscopic findings, we characterized the findings based on the PDAI,^2,3^ following review of images and reports into endoscopic findings of inflammation that include “erythema/edema,” “erosions/friability,” “ulceration,” “stenosis,” “granularity,” and “loss of vascular pattern.” While the Chicago classification study did not use “mucous exudates” to classify the phenotype and we followed this method,^11^ this finding was included in the calculation of mPDAI. Features of perianal, anal, or perineal disease included anal fissures, fistulas, skin tags, or hemorrhoids. We reviewed all available reports of pouchoscopies after ileostomy takedown and characterized pouch phenotypes based on the endoscopy report and images. If the endoscopic description was not explicit and the findings were not noted, the endoscopy images were used to report the findings. If the endoscopic description and images were not available, we assigned these data as “not available.” We classified the pouches into 7 main pouch phenotypes based on the anatomic location of abnormalities: (a) normal, (b) AL involvement, (c) IL involvement, (d) diffuse inflammation of the pouch body, (e) focal inflammation of the pouch body, (f) cuffitis, and (g) pouch fistulas (now called “pouch-related fistulas” for clarification).^11^

A “normal pouch” was defined as the pouch without any abnormal endoscopic findings at all anatomical locations of the J pouch, anastomosis, cuff, anal canal, and perianal area, and any type of fistulas. AL or IL involvement was defined as a patient with any type of endoscopic inflammation in the AL or IL, respectively. In this analysis, “pouchitis” was defined as one or more endoscopic findings of inflammation in any of the tip, proximal, or distal pouch. Diffuse inflammation of the pouch body was defined as two or more endoscopic findings of inflammation in all anatomical locations of the pouch body (the tip, proximal, and distal pouch). Focal inflammation of the pouch body was defined as pouchitis that did not meet the criteria for diffuse inflammation of the pouch body. Cuffitis was defined as a pouch with any type of endoscopic inflammation in the rectal cuff. Pouch-related fistulas included pouches with any type of fistulas arising from the pouch, the rectal cuff, or the anal/perineal area noted by pouchoscopy or other imaging studies including pouchogram, computed tomography, or magnetic resonance imaging after 6 months from ileostomy takedown, in order to exclude fistulas that occur temporally proximate to surgery as a technical complication.^10^

In order to isolate symptoms related to specific phenotypes, we attempted to restrict this analysis to patients who had a pouchoscopy identifying a phenotype. However, given the limited number of scopes with isolated AL involvement, IL involvement, and pouch-related fistulas, we included patients with these phenotypes accompanied by focal inflammation of the pouch as well. To clarify phenotype-related symptoms, patients with anastomotic involvement including stricture were excluded. Patients with perianal/anal involvement were excluded except for an analysis of pouch-related fistulas. The mPDAI was assessed based on endoscopic findings and clinical symptoms at the clinical visit closest to the date of the pouchoscopy. The endoscopic subscore of the mPDAI was evaluated at the site of inflammation. With reference to the Endoscopic Pouch Score,^13^ the endoscopic subscore of the mPDAI was calculated not only inside but also outside of the pouch body (eg, AL, IL, and cuff). Regarding clinical symptoms, we included “stool frequency,” “rectal bleeding,” “fecal urgency or abdominal cramps,” “fever >37.8 °C,” “perianal/anal pain or discomfort,” “incomplete emptying,” and “incontinence.” If medical records did not specifically report a symptom, we registered “not available” and conducted complete-case analysis in which only available data are assessed. To calculate mPDAIs, we only used available data as well.

Study data were collected and managed using REDCap electronic data capture tools hosted at the University of Chicago.^14^

### Statistical Analysis

Kruskal-Wallis test was used to assess the overall difference in subscores and total scores of mPDAIs between phenotypes. As a post hoc analysis, Steel-Dwass test was used to compare the scores of all pairs of groups. *P* values < .05 were considered statistically significant. Data were analyzed by EZR (Saitama Medical Center, Jichi Medical University, Saitama, Japan),^15^ which is a graphical user interface for R (The R Foundation for Statistical Computing, version 2.13.0, Vienna, Austria).

### Ethical Considerations

All procedures performed in studies involving human participants were in accordance with the ethical standards of the institutional and/or national research committee and with the 1964 Helsinki Declaration and its later amendments or comparable ethical standards. Since this study retrospectively analyzes existing clinical data and does not involve the collection of new samples, the requirement for informed consent was waived. The study was approved by the Institutional Review Board of the University of Chicago (IRB numbers 16-0061 and 15573A).

## Results

### Patient Characteristics

We identified 103 IBD patients for this analysis (Figure 1). The demographic characteristics of the patients and their postoperative treatments are summarized in Tables 1 and 2. Of these patients, 57 were men (55.3%) and 91 were White (89.2%). Included patients had a preoperative diagnosis of UC (94; 91.3%), indeterminate colitis (8; 7.8%), and Crohn’s disease (1; 1.0%). The majority of patients (83.5%) were diagnosed with IBD when they were 18 years or older. As for surgical technique, a 3-stage IPAA was performed in 49 patients (52.1%). A staple anastomosis and a hand-sewn anastomosis were selected in 57 patients (69.5%) and 25 patients (30.5%), respectively. The number of patients with PSC was 5 (4.9%) in this cohort (Table 1). With regard to the postoperative treatments, 44.7% of patients used antidiarrheal medications including loperamide or diphenoxylate-atropine. Ciprofloxacin and metronidazole were used in 15 patients (14.6%) and 12 patients (11.7%), respectively. More than 90% of patients were not treated with other medications including mesalamine, immunomodulators, steroids, or biologics/small-molecule agents (antitumor necrosis factor drugs, vedolizumab, ustekinumab, or tofacitinib; Table 2). The median duration between the date of clinical visit and the date of the pouchoscopy was 1 month (interquartile range [IQR] 0-2 months).

**Table 1.** Demographic characteristics.

Demographics	Group	Included patients (*N* = 103)
Sex (%)	Female	46 (44.7)
	Male	57 (55.3)
White (%)	No	11 (10.8)
	Yes	91 (89.2)
Age at diagnosis (%)	<18 years	17 (16.5)
	≥18 years	86 (83.5)
Age at colectomy (%)	<18 years	7 (6.8)
	≥18 years	96 (93.2)
BMI (%)	<25	50 (51.0)
	≥25	48 (49.0)
Disease duration (%)	<7 years	63 (61.2)
	≥7 years	40 (38.8)
Dysplasia/Cancer (%)	No	85 (85.9)
(Surgical indication)	Yes	14 (14.1)
Extensive colitis (%)	E1/2	15 (16.3)
	E3	77 (83.7)
Ever smoker (%)	No	79 (77.5)
	Yes	23 (22.5)
Family history of IBD (%)	No	67 (69.8)
	Yes	29 (30.2)
Technique of IPAA (%)	Stapled	57 (69.5)
	Hand-sewn	25 (30.5)
Three-stage IPAA (%)	No	45 (47.9)
	Yes	49 (52.1)
Preoperative CDI (%)	No	82 (86.3)
	Yes	13 (13.7)
Preoperative UC (%)	No	9 (8.7)
	Yes	94 (91.3)
PSC (%)	No	98 (95.1)
	Yes	5 (4.9)

Abbreviations: BMI, body mass index; IBD, inflammatory bowel disease; IPAA, ileal pouch-anal anastomosis; CDI, *Clostridioides difficile* infection; UC, ulcerative colitis; PSC, primary sclerosing cholangitis.

**Table 2.** Postoperative treatments.

Postoperative treatments	Group	Included patients(*N* = 103)
Loperamide or diphenoxylate-atropine (%)	No	57 (55.3)
	Yes	46 (44.7)
Ciprofloxacin (%)	No	88 (85.4)
	Yes	15 (14.6)
Metronidazole (%)	No	91 (88.3)
	Yes	12 (11.7)
Anti-TNF drugs (%)	No	96 (93.2)
	Yes	7 (6.8)
Immunomodulators (%)	No	97 (94.2)
	Yes	6 (5.8)
Oral budesonide (%)	No	98 (95.1)
	Yes	5 (4.9)
VSL#3 (%)	No	99 (96.1)
	Yes	4 (3.9)
Oral steroid (%)	No	100 (97.1)
	Yes	3 (2.9)
Topical mesalamine (%)	No	100 (97.1)
	Yes	3 (2.9)
Oral mesalamine (%)	No	101 (98.1)
	Yes	2 (1.9)
Topical steroid (%)	No	101 (98.1)
	Yes	2 (1.9)
Vedolizumab (%)	No	102 (99.0)
	Yes	1 (1.0)
Tacrolimus (%)	No	102 (99.0)
	Yes	1 (1.0)

Abbreviation: TNF, tumor necrosis factor.

### Endoscopic Phenotype

In this cohort, there were 25 patients with normal pouch (24.3%), 4 patients (3.9%) with AL involvement, 14 (13.6%) with IL involvement, 7 (6.8%) with diffuse inflammation, 25 (24.3%) with focal inflammation of the pouch body, 18 (17.5%) with cuffitis, and 10 (9.7%) with pouch-related fistulas (Table 3). Focal inflammation of the pouch body was overlapped in 4 patients with AL involvement, 11 patients with IL involvement, and 3 patients with pouch-related fistulas. All of 3 patients with IL involvement as a single phenotype had IL stenosis (Table 3). Among 10 patients with pouch-related fistulas, perianal fistulas, rectovaginal/anovaginal fistulas, and enterocutaneous fistulas were identified in 10 patients (100%), 2 patients (20%), and 1 patient (10%), respectively.

**Table 3.** The frequency of each endoscopic phenotype as a single phenotype.

Phenotypes	Group	Included patients *N* = 103
Normal (%)	Single phenotype	25 (24.3)
Afferent limb involvement (%)	With focal inflammation	4 (3.9)
Inlet involvement (%)	With focal inflammation	11 (10.7)
	Single phenotype (inlet stenosis)	3 (2.9)
Diffuse inflammation (%)	Single phenotype	7 (6.8)
Focal inflammation (%)	Single phenotype	25 (24.3)
Cuffitis (%)	Single phenotype	18 (17.5)
Pouch-related fistulas (%)	Single phenotype	7 (6.8)
	With focal inflammation	3 (2.9)

### Clinical Symptoms

In our complete-case analysis, “usual stool frequency” or “none/rare rectal bleeding” were observed in half or more patients with normal pouch, IL involvement, diffuse inflammation, focal inflammation of the pouch body, or pouch-related fistulas. In contrast, patients with cuffitis, and AL involvement often experienced “more stool frequency than usual” (66.7% and 75%, respectively) or “rectal bleeding” (31.2% and 100%, respectively; Table 4). In terms of “fecal urgency/abdominal cramps,” 57% of patients with normal pouch had these symptoms, whereas more than half of patients with focal inflammation, and those with cuffitis occasionally or usually experienced these symptoms. In our analysis, only one patient with IL involvement had “fever >37.8 °C” (Table 4).

**Table 4.** The rate of clinical symptoms in mPDAI among endoscopic phenotypes.

Symptoms in mPDAI	Group	Normal (*N* = 25)	AL (*N* = 4)	IL (*N* = 14)	Diffuse (*N* = 7)	Focal (*N* = 25)	Cuffitis (*N* = 18)	Fistulas (*N* = 10)	*P*-value^*^
Stool frequency (%)	0: Usual	19 (76.0)	1 (25.0)	7 (50.0)	3 (50.0)	16 (64.0)	6 (33.3)	6 (75.0)	.117
	1: 1-2 stools/day > usual	4 (16.0)	2 (50.0)	2 (14.3)	1 (16.7)	5 (20.0)	3 (16.7)	0 (0.0)	
	2: 3 or more stools > usual	2 (8.0)	1 (25.0)	5 (35.7)	2 (33.3)	4 (16.0)	9 (50.0)	2 (25.0)	
Rectal bleeding (%)	0: None or rare	13 (92.9)	0 (0.0)	8 (88.9)	3 (100.0)	14 (100.0)	11 (68.8)	3 (75.0)	.039
	1: Present daily	1 (7.1)	1 (100.0)	1 (11.1)	0 (0.0)	0 (0.0)	5 (31.2)	1 (25.0)	
Fecal urgency or abdominal cramps (%)	0: None	6 (42.9)	2 (66.7)	5 (55.6)	3 (75.0)	6 (40.0)	6 (46.2)	3 (60.0)	.926
	1: Occasional	5 (35.7)	1 (33.3)	2 (22.2)	0 (0.0)	6 (40.0)	6 (46.2)	1 (20.0)	
	2: Usual	3 (21.4)	0 (0.0)	2 (22.2)	1 (25.0)	3 (20.0)	1 (7.7)	1 (20.0)	
Fever > 37.8 °C (%)	0: Absent	23 (100.0)	3 (100.0)	10 (90.9)	5 (100.0)	22 (100.0)	15 (100.0)	8 (100.0)	.322
	1: Present	0 (0.0)	0 (0.0)	1 (9.1)	0 (0.0)	0 (0.0)	0 (0.0)	0 (0.0)	

^*^Chi-square test.

Abbreviations: AL, afferent limb; IL, inlet; mPDAI, modified pouchitis disease activity index.

With regard to “perianal/anal pain or discomfort,” 50% or more patients with not only inflammatory phenotypes but also normal pouch experienced these symptoms. In particular, 80% or more patients with cuffitis, pouch-related fistulas, and AL involvement had these symptoms. Although data regarding “incomplete emptying” is sometimes missed, 67% of patients (2/3) with normal pouch did not have this symptom. Conversely, among 6 patients with cuffitis who had available data, 6 patients (100%) experienced “incomplete emptying.” As for “incontinence,” 23% of patients with normal pouch described this symptom, while half or more patients with AL involvement, IL involvement, and pouch-related fistulas described this symptom (Table 5).

**Table 5.** The rate of clinical symptoms which are not included in mPDAI among endoscopic phenotypes.

Clinical symptoms	Group	Normal (*N* = 25)	AL (*N* = 4)	IL (*N* = 14)	Diffuse (*N* = 7)	Focal (*N* = 25)	Cuffitis (*N* = 18)	Fistulas (*N* = 10)	*P*-value^*^
Perianal/anal pain or discomfort (%)	No	6 (46.2)	0 (0.0)	1 (33.3)	1 (50.0)	3 (25.0)	2 (13.3)	2 (20.0)	.530
	Yes	7 (53.8)	1 (100.0)	2 (66.7)	1 (50.0)	9 (75.0)	13 (86.7)	8 (80.0)	
Incomplete emptying (%)	No	2 (66.7)	0 (—)	1 (100.0)	0 (—)	2 (100.0)	0 (0.0)	0 (—)	NaN
	Yes	1 (33.3)	0 (—)	0 (0.0)	0 (—)	0 (0.0)	6 (100.0)	0 (—)	
Incontinence (%)	No	10 (76.9)	1 (33.3)	2 (50.0)	2 (66.7)	10 (76.9)	7 (58.3)	1 (50.0)	.703
	Yes	3 (23.1)	2 (66.7)	2 (50.0)	1 (33.3)	3 (23.1)	5 (41.7)	1 (50.0)	

^*^Chi-square test.

Abbreviations: AL, afferent limb; IL, inlet; mPDAI, modified pouchitis disease activity index; NaN, Not a Number.

### Modified PDAI

**Figure 2.**
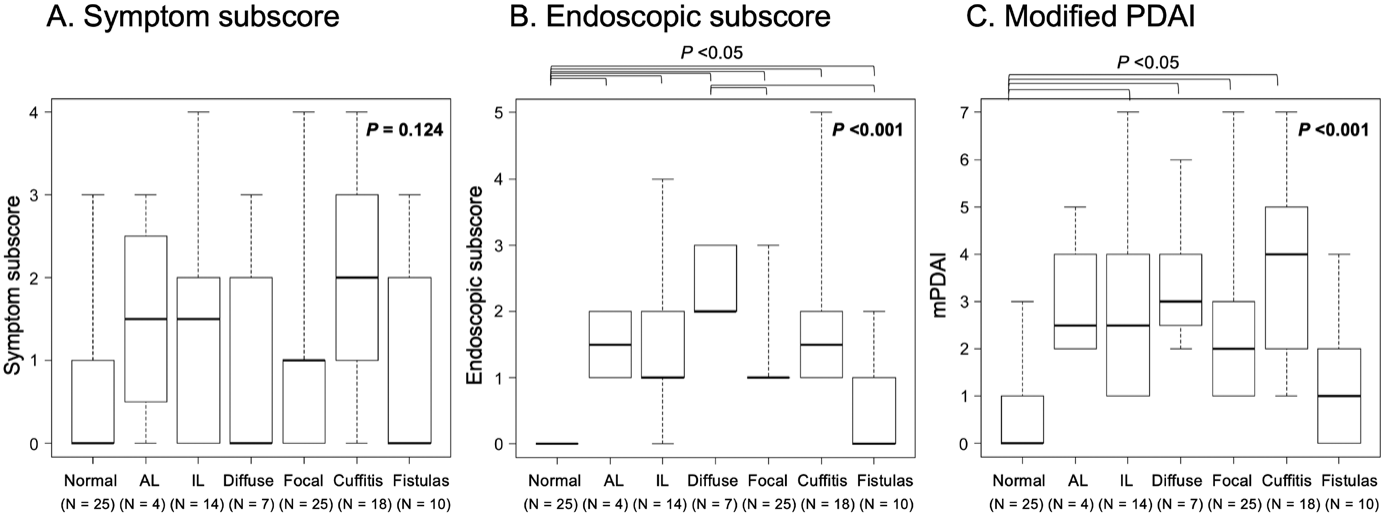
A, Box and whisker plots of symptom scores in the modified pouchitis disease activity index (mPDAI). A box represents an interquartile range, and a thick horizontal line shows a median value. Whiskers are extended to minimum and maximum values. B, Box and whisker plots of endoscopic scores in mPDAI. A box represents an interquartile range, and a thick horizontal line shows a median value. Whiskers are extended to minimum and maximum values. C, Box and whisker plots of the mPDAI. A box represents an interquartile range, and a thick horizontal line shows a median value. Whiskers are extended to minimum and maximum values. Kruskal-Wallis test is used to assess the overall difference in subscores and total scores of mPDAIs between phenotypes. As a post hoc analysis, Steel-Dwass test is used to compare these scores of all pairs of groups. *P* values < .05 are considered statistically significant. Upper brackets indicated the difference between two groups was significant. AL, afferent limb; IL, inlet.

We initially assessed the subscores of clinical symptoms and endoscopic scores then calculated mPDAI in each phenotype. The median subscore of clinical symptoms was 0 (IQR 0-1.0) in patients with a normal pouch. Meanwhile, the highest median subscore of clinical symptoms was 2.0 (IQR 1.0-3.0) in patients with cuffitis, followed by 1.5 (IQR 0.75-2.25) in AL involvement, 1.5 (IQR 0-2.0) in IL involvement, 1.0 (IQR 0-1.0) in focal inflammation, 0 (IQR 0-2.0) in diffuse inflammation, and 0 (IQR 0-1.75) in pouch-related fistulas. Kruskal-Wallis test showed that the overall difference in subscores of clinical symptoms between phenotypes was not significant (*P* = .124; Figure 2A).

The median endoscopic subscore is 0 (IQR 0-0) in patients with a normal pouch. Among patients with inflammatory phenotypes, the highest median endoscopic subscore was 2.0 (IQR 2.0-3.0) in patients with diffuse inflammation, followed by 1.5 (IQR 1.0-2.0) in those with cuffitis, 1.5 (IQR 1.0-2.0) in AL involvement, 1.0 (IQR 1.0-2.0) in those with IL involvement, 1.0 (IQR 1.0-1.0) in those with focal inflammation, and 0 (IQR 0-1.0) in those with pouch-related fistulas. Kruskal-Wallis test showed that the overall difference in endoscopic subscore between phenotypes was significant (*P* < .001). The post hoc analysis showed that the endoscopic subscore in the normal pouch was significantly lower than that in each of the inflammatory phenotypes (*P* < .05). Meanwhile, the endoscopic subscore in those with diffuse inflammation was significantly higher compared with those with focal inflammation, and pouch-related fistulas (*P* = .004, and *P* = .025, respectively; Figure 2B).

The median mPDAI was 0 (IQR 0-1.0) in patients with normal pouch. In patients with inflammatory phenotypes, the highest median mPDAI was 4.0 (IQR 2.25-4.75) in patients with cuffitis, followed by 3.0 (IQR 2.5-4.0) in those with diffuse inflammation, 2.5 (IQR 1.25-4.0) in those with IL involvement, 2.5 (IQR 2.0-3.5) in those with AL involvement, 2.0 (IQR 1.0-3.0) in those with focal inflammation, and 1.0 (IQR 0.25-2.0) in those with pouch-related fistulas. Kruskal-Wallis test showed that the overall difference in mPDAI between phenotypes was significant (*P* < .001). The post hoc analysis showed that mPDAI in those with a normal pouch was significantly lower in comparison to those with IL involvement, diffuse inflammation, focal inflammation, or cuffitis (*P* < .05; Figure 2C).

### Longitudinal Analysis of Patients Who Have Achieved Pouch Normalization

We identified 7 patients with a single phenotype have achieved subsequent pouch normalization (Supplementary Table 2). The median clinical symptom subscore was not significantly different before and after pouch normalization (*P* = .59), whereas the endoscopic subscore was significantly improved after pouch normalization (*P* = .048). As a result, mPDAI numerically decreased after the pouch normalization (*P* = .053; Supplementary Figure S1).

## Discussion

Using the Chicago classification of pouchitis, we have demonstrated that mPDAI in an endoscopically normal pouch was the lowest among all of the endoscopic phenotypes and mPDAIs differed among the inflammatory phenotypes of pouchitis.^11^ Our analysis found that patients with inflammatory phenotypes, particularly cuffitis or AL involvement, were more likely to have “more stool frequency than usual” or “rectal bleeding” compared to those with a normal pouch. In contrast, more than half of patients with a normal pouch had “fecal urgency/abdominal cramps.” All these findings suggested that “stool frequency” or “rectal bleeding” can be associated with inflammatory conditions, especially in the rectal cuff or AL, but “fecal urgency/abdominal cramps” may not be specific for any phenotype. Indeed, the subscore of clinical symptoms in mPDAI as well as total mPDAI in patients with cuffitis were the highest among all phenotypes, suggesting that mPDAI has a limited utility to differentiate clinical symptoms of cuffitis from other inflammatory phenotypes. Our findings demonstrated that inflammation in the rectal cuff can increase subscore of clinical symptoms in mPDAI and patients with a high score of mPDAI did not necessarily have endoscopic pouch inflammation.

Recently, the importance of evaluating inflammatory findings according to the anatomical location of the pouch is noted. For instance, the consensus guideline from the International Ileal Pouch Consortium states that at least three biopsies are taken from the anal transition zone or cuff, along with biopsies from the AL and pouch body during the surveillance pouchoscopy.^16^ In addition, the Endoscopic Pouch Score has recently been reported, using segmental scoring similar to the Simple Endoscopic Score for Crohn’s Disease.^17^ Importantly, this scoring evaluates inflammation at the pouch body, AL, inlet, and cuff as well as stricture at the ileoanal anastomosis.^13^ Our findings also suggest that documenting the location of endoscopic findings may be important. Furthermore, measures of clinical disease activity of the pouch and cuff should be distinct^18^ and may need to establish distinct score systems that are specified for each endoscopic phenotype.

Clinical symptoms of mPDAI also include “fever >37.8 °C.” However, we identified only one case with this symptom, in a patient with IL involvement, suggesting that this symptom might be omitted when scoring mPDAI. Furthermore, our analysis suggested that mPDAI did not accurately capture disease activity in patients with pouch-related fistulas because its mPDAI was the lowest among patients with inflammatory phenotypes. To identify better candidates for clinical disease monitoring of inflammatory phenotypes, we also assessed clinical symptoms that the mPDAI does not include. Our analysis found that “perianal/anal pain or discomfort” are common symptoms among all endoscopic phenotypes including those with a normal pouch. However, patients with cuffitis and those with pouch-related fistulas were more likely to have these symptoms (≥80%) compared with those with normal pouch (54%) or other phenotypes (50%-75%). Moreover, although some data of “incomplete emptying” were incomplete, our complete-case analysis showed all patients with the phenotype of cuffitis had this symptom. These findings suggest that “perianal/anal pain or discomfort” or “incontinence” can be symptoms that should be included in the assessment of clinical disease activity for cuffitis or pouch-related fistulas. Given the rate of “incontinence” in patients with a normal pouch (23%) was lower than that of inflammatory phenotypes except for focal inflammation (33%-67%), this symptom may be a bit more specific for inflammatory phenotypes as well, although is not specific enough to separate patients a priori without endoscopic evaluation. Interestingly, we also found that “incontinence” was frequently observed in patients with AL involvement (67%), IL involvement (50%), and pouch-related fistulas (50%). Given these phenotypes are included in the commonly accepted definition of Crohn’s disease of the pouch,^19^ “incontinence” may be explored further as a candidate for the measure of clinical disease activity in patients with Crohn’s disease-like pouch phenotype.

We found a discrepancy between clinical symptoms and disease extent of the pouch body in this study. Expectedly, the endoscopic subscore of mPDAI in patients with diffuse inflammation as a single phenotype was the highest among all of phenotypes. However, the median score of clinical symptoms in patients with focal inflammation [1 (IQR 0-1)] was higher than that in patients with diffuse inflammation of the pouch body [0 (IQR 0-2)], suggesting that extensive inflammation of the pouch body may not necessarily be correlated with the severity of clinical symptoms. Consistent with our findings, Kayal et al.^8^ demonstrated that mucosal breaks were present in nearly a quarter of asymptomatic patients with the pouch. While it remains unclear whether patients with diffuse pouchitis with fewer symptoms are at risk for future progression to symptomatic pouchitis or development of colorectal neoplasia, a recent retrospective study showed that patients with diffuse inflammation of the pouch body had a significantly increased risk of chronic pouchitis,^20^ which is a purported risk factor for pouch neoplasia.^21^ Moreover, since diffuse inflammation often overlaps with other phenotypes (eg, IL involvement and cuffitis) and deteriorates long-term pouch outcome,^22^ patients may have pouchitis-related symptoms when diffuse inflammation is overlapped with other phenotypes. Therefore, we suggest that careful endoscopic monitoring of diffuse inflammation would be preferred. Although limited utility of PDAI for the assessment of clinical disease activity of pouchitis, we also found that patients with normal pouch had the lowest value of mPDAI, suggesting that mPDAI may reflect pouch normalization and be a negative predictor of inflammation of the pouch. In fact, our longitudinal analysis showed that mPDAI improved after achieving pouch normalization. Further studies with larger sample sizes are needed to confirm our findings.

There are several strengths and limitations in this study. It is a significant strength that this study is the first to compare mPDAIs among endoscopic phenotypes of the pouch. To extract data regarding clinical symptoms, we initially identified a scope that showed a single phenotype and then reviewed clinical symptoms at the clinical visit closest to the scope date, although analyses of AL involvement, IL involvement, and pouch-related fistulas needed to include focal inflammation due to the limited number of these single phenotypes. Since patients with focal inflammation were less likely to have stool frequency more than usual (36%) and rectal bleeding (0%) compared with other inflammatory phenotypes, we believe that some of our data reflect clinical symptoms directly caused by a single phenotype. Although pouchoscopy is performed according to the standard operating protocol and providers dedicatedly describe the medical records focusing on pouchitis-related symptoms in our center, this study is retrospective in nature and missing data could not be ignored. Hence, we approached our analysis based on complete-case analysis which removed missing data. Given that 45% of patients were treated with antidiarrheal drugs, clinical symptoms such as “stool frequency” may be affected by these medications. However, the rate of patients who were exposed to other anti-inflammatory medications was less than 15%, suggesting that there were a few patients who required treatments to control their symptoms in this study. Our study is also limited to a single tertiary center and the number of each phenotype, particularly AL involvement, was small. Hence, prospective and multicenter studies with larger sample size are needed to confirm our findings.

## Conclusions

We compared mPDAIs among endoscopic phenotypes of the pouch and found that patients with normal pouch had the lowest mPDAI and those with cuffitis had the highest mPDAI in our cohort. Our findings suggest that mPDAI may not be useful in the current era because it does not assess all phenotypes well. Monitoring of clinical disease activity for patients with cuffitis should be distinct from other inflammatory phenotypes and may need indices including clinical symptoms which are more specific for cuffitis (eg, “perianal/anal pain or discomfort” or “incomplete emptying”). Further prospective studies with larger sample size are needed to confirm our findings and to understand if mPDAI can decrease after achieving pouch normalization, which would suggest that such normalization can be a valid therapeutic target when treating patients with J pouches.

## Supplementary Material

otae045_suppl_Supplementary_Tables

otae045_suppl_Supplementary_Figure_S1

otae045_suppl_Supplementary_Figure_Legend

## Data Availability

The data underlying this article will be shared upon request from the corresponding author.
