# Supplementary material for: A Comparative Analysis of Clinical Symptoms and Modified Pouchitis Disease Activity Index Among Endoscopic Phenotypes of the J Pouch in Patients With Inflammatory Bowel Disease
Source: Crohns Colitis 360. 2024 Aug 2;6(3):otae045. doi: 10.1093/crocol/otae045 (PMC11438232; doi:10.1093/crocol/otae045)
Supplement: otae045_suppl_Supplementary_Tables [file otae045_suppl_supplementary_tables.docx]

**Supplementary Table 1. Data collected from medical and endoscopic records**

| Medical records | Medical records | Endoscopic records |
| --- | --- | --- |
| *(Demographics data)* | *(Pouchitis-related symptoms)* |  |
| Gender | Stool frequency | Erythema/edema |
| Race | Rectal bleeding | Erosions/friability |
| Age at diagnosis | Fecal urgency or abdominal cramps | Ulceration |
| Age at colectomy | Fever >37.8°C | Stenosis |
| Body mass index | Perianal/anal pain or discomfort | Granularity |
| Disease duration | Incomplete emptying | Loss of vascular pattern |
| Surgical indication | Incontinence | fistulas |
| Disease extent  (Montreal classification) |  | Anal fissures |
| Smoking status |  | Skin tags |
| Family history of IBD |  | Hemorrhoids |
| Technique of IPAA |  | Mucous exudate |
| Number of stages of IPAA |  |  |
| Preoperative CDI |  |  |
| Preoperative diagnosis |  |  |
| PSC |  |  |
| Preoperative diagnosis |  |  |
| PSC |  |  |
| Postoperative therapies at the time when clinical symptoms were evaluated |  |  |

BMI, body mass index; IBD, inflammatory bowel disease; IPAA, ileal-pouch anal anastomosis;

CDI, *Clostridioides difficile* infection; UC, ulcerative colitis; PSC, primary sclerosing cholangitis

**Supplementary Table 2. Patients with a single inflammatory phenotype who have achieved subsequent pouch normalization (n = 7)**

| **Phenotypes** | **Group** | **Included patients** |
| --- | --- | --- |
|  |  | **N = 7** |
| Afferent limb involvement (%) | With focal inflammation | 0 (0) |
| Inlet involvement (%) | Single phenotype  (IL stenosis) | 1 (14%) |
| Diffuse inflammation (%) | Single  phenotype | 0 (0) |
| Focal inflammation (%) | Single  phenotype | 2 (28%) |
| Cuffitis (%) | Single  phenotype | 3 (42%) |
| Pouch-related fistulas (%) | Single  phenotype | 1 (14%) |
