# Supplementary figures and images for: A Comparative Analysis of Clinical Symptoms and Modified Pouchitis Disease Activity Index Among Endoscopic Phenotypes of the J Pouch in Patients With Inflammatory Bowel Disease
Source: Crohns Colitis 360. 2024 Aug 2;6(3):otae045. doi: 10.1093/crocol/otae045 (PMC11438232; doi:10.1093/crocol/otae045)

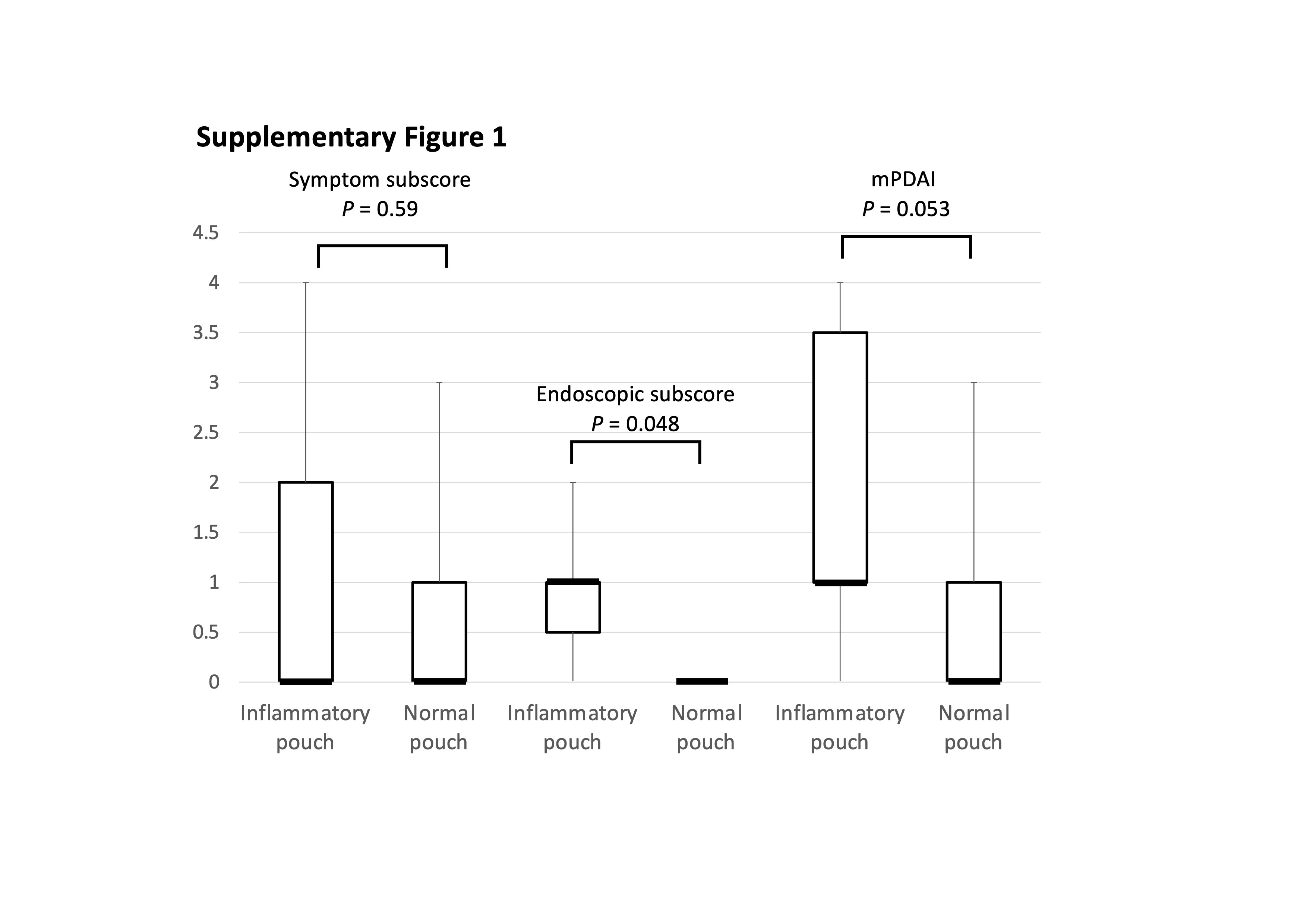

Supplement: otae045_suppl_Supplementary_Figure_S1 [file otae045_suppl_supplementary_figure_s1.jpeg]
