# Supplementary material for: A Comparative Analysis of Clinical Symptoms and Modified Pouchitis Disease Activity Index Among Endoscopic Phenotypes of the J Pouch in Patients With Inflammatory Bowel Disease
Source: Crohns Colitis 360. 2024 Aug 2;6(3):otae045. doi: 10.1093/crocol/otae045 (PMC11438232; doi:10.1093/crocol/otae045)
Supplement: otae045_suppl_Supplementary_Figure_Legend [file otae045_suppl_supplementary_figure_legend.docx]

**Supplementary Figure Legend**

**Supplementary Figure 1.** Longitudinal analysis of patients with an inflammatory single phenotype who have subsequently achieved pouch normalization (n = 7). This analysis compares the symptom and endoscopic subscores of the modified pouchitis disease activity index (mPDAI) and its total score before and after pouch normalization. Wilcoxon signed-rank test is used to assess the difference in subscores and total scores of mPDAIs before and after pouch normalization.
